# Cochlear-implant Mandarin tone recognition with a disyllabic word corpus

**DOI:** 10.3389/fpsyg.2022.1026116

**Published:** 2022-10-17

**Authors:** Xiaoya Wang, Yefei Mo, Fanhui Kong, Weiyan Guo, Huali Zhou, Nengheng Zheng, Jan W. H. Schnupp, Yiqing Zheng, Qinglin Meng

**Affiliations:** ^1^The First Clinical Medical College of Jinan University, Guangzhou, China; ^2^Department of Otolaryngology, Guangzhou Women and Children's Medical Center, Guangzhou, China; ^3^Acoustics Laboratory, School of Physics and Optoelectronics, South China University of Technology, Guangzhou, China; ^4^The Guangdong Key Laboratory of Intelligent Information Processing, College of Electronics and Information Engineering, Shenzhen University, Shenzhen, China; ^5^Department of Hearing and Speech Science, Xin Hua College of Sun Yat-sen University, Guangzhou, China; ^6^Department of Biomedical Sciences and Department of Neuroscience, City University of Hong Kong, Hong Kong, Hong Kong SAR, China; ^7^Department of Otolaryngology, Sun Yat-sen Memorial Hospital, Sun Yat-sen University, Guangzhou, China

**Keywords:** cochlear implants, Mandarin tone, pitch contour, loudness contour, lexical tone

## Abstract

Despite pitch being considered the primary cue for discriminating lexical tones, there are secondary cues such as loudness contour and duration, which may allow some cochlear implant (CI) tone discrimination even with severely degraded pitch cues. To isolate pitch cues from other cues, we developed a new disyllabic word stimulus set (Di) whose primary (pitch) and secondary (loudness) cue varied independently. This Di set consists of 270 disyllabic words, each having a distinct meaning depending on the perceived tone. Thus, listeners who hear the primary pitch cue clearly may hear a different meaning from listeners who struggle with the pitch cue and must rely on the secondary loudness contour. A lexical tone recognition experiment was conducted, which compared Di with a monosyllabic set of natural recordings. Seventeen CI users and eight normal-hearing (NH) listeners took part in the experiment. Results showed that CI users had poorer pitch cues encoding and their tone recognition performance was significantly influenced by the “missing” or “confusing” secondary cues with the Di corpus. The pitch-contour-based tone recognition is still far from satisfactory for CI users compared to NH listeners, even if some appear to integrate multiple cues to achieve high scores. This disyllabic corpus could be used to examine the performance of pitch recognition of CI users and the effectiveness of pitch cue enhancement based Mandarin tone enhancement strategies. The Di corpus is freely available online: https://github.com/BetterCI/DiTone.

## 1. Introduction

Linguists define “lexical tone” as the phenomenon when two syllables which differ in their pitch contour but are otherwise identical can have different meanings. Mandarin Chinese is a tonal language in which each syllable has four typical tones, each has a characteristic pitch contour. By convention, Tone 1 has a high-flat pitch, Tone 2 a rising pitch, Tone 3 is falling-then-rising in a relatively low pitch range, and Tone 4 has a falling pitch. Linguistic meaning can be distinguished by these four tones. The register and range of pitch contours vary among utterances and persons. Psychoacoustical studies have shown that pitch-related acoustic cues are complex and manifest within multiple features in both temporal and spectral domains of sounds (Schnupp et al., 2011; Oxenham, 2018). Normal hearing (NH) listeners of tonal languages can use pitch cues to distinguish lexical tones robustly even when acoustic signals are degraded by environmental noise, low-fidelity playback, human speech production variability, etc. In contrast, for most cochlear implant (CI) recipients, lexical tone perception is still challenging (Lu et al., 2022), and performance varies significantly across recipients and in environments (Chang et al., 2016; Liu et al., 2017; Mao and Xu, 2017; Li et al., 2018; Tang et al., 2019). This is perhaps unsurprising given CI recipients' weaker and more variable abilities to extract pitch cues from acoustic signals (Tao et al., 2015; Mok et al., 2017; Vandali et al., 2017). Limitations in pitch extraction can occur on multiple stages of the CI supplied auditory system, from the device's signal processing strategy through peripheral auditory neural processing, all the way to auditory cortical processing and cognition (Zhang, 2019; Zhou et al., 2022).

However, speech researchers have long recognized that pitch cues are not the only acoustic cues that could be used for lexical tone discrimination. Secondary cues, such as amplitude contour (Whalen and Xu, 1992; Kuo et al., 2008), duration (Fu and Zeng, 2000; Xu et al., 2002; Yang et al., 2017), and spectral (timbre) contour (Liang, 1963), tend to covary with the pitch cues and may be useful when pitch cues are significantly degraded. Thus, loudness and timbre can occasionally serve as alternative cues in tasks which are classically thought of as pitch-dependent, including lexical tone and musical melody perception, and this has been observed in both NH and, more strongly, in CI listeners (McDermott et al., 2008; Cousineau et al., 2010; Luo et al., 2014, 2019). Manipulating the timbre contour for tone enhancement in speech is problematic since changing the spectral shape would likely affect the formant structure of the manipulated speech. In contrast, the amplitude contour could be manipulated to co-vary more strongly with the fundamental frequency (F0) contour to facilitate Mandarin tone perception with CIs (Luo and Fu, 2004; Kim et al., 2021), and some studies indicated that these kinds of strategies can be effective in actual CI users (Ping et al., 2017; Meng et al., 2018).

The confounds created by co-varying pitch and non-pitch cues to the Mandarin tone also imply that previous Mandarin tone recognition experiments with CI participants, which simply used naturally recorded speech stimuli, will have measured the ability to utilize some combination of several types of acoustic cues. These experiments therefore cannot give an independent estimate of the CI user's ability to use specifically pitch cues to discriminate lexical tones. Indeed, secondary cues can be quite reliable and could be strong enough to lead to ceiling effects in tone identification. This could perhaps explain why some previous tests of lexical tone enhancement strategies found no or only little improvement (Han et al., 2009; Vandali et al., 2017).

Pitch and duration cues for lexical tone perception have been studied by Peng et al. (2009, 2017). They orthogonally manipulated F0 (pitch) contour, intensity (loudness) contour, and duration, to study how the interaction between these cues influence the perception of English intonation (Peng et al., 2009) or Chinese lexical tone (Peng et al., 2017). Covarying cues generally caused better results than conflicting cues for CI listeners, but no significant difference was found for NH listeners. In the tone perception study by Peng et al. (2017), the pitch contour and duration of the second syllable /jing/ in the disyllabic word /yǎn jing/ were manipulated to generate two alternative meanings: /yǎn jīng/ (Tone 1) means eyes, and /yǎn jìng/ (Tone 4) means eyeglasses. Using disyllables rather than monosyllables for tests of this nature is preferable because Chinese monosyllables tend to have many homophonic meanings, while the meaning of disyllables tends to be much more unambiguously determined by the tone, creating less uncertainty in the participants' mind. While Peng et al. (2017) did study pitch and duration cues for lexical tone, they did not investigate the role of the amplitude contour, even though this is a powerful secondary cue.

In order to dissociate the contributions of pitch and non-pitch cues to tone recognition, we developed a set of Mandarin syllables where the pitch cues of target tones vary independently of secondary loudness and duration cues. This was inspired by Peng et al. (2009, 2017). In our preliminary study (Meng et al., 2018), we manipulated the pitch contour and the loudness contour of the second syllable /shi/ of a disyllable /lǎo shi/ independently to generate speech sounds that could be interpreted to convey one of three possible word meanings: /lǎo shī/ (Tone 1) means “teacher”, /lǎo shí/ (Tone 2) means “well-behaved”, and /lǎo shì/ (Tone 4) means “always”. Different weighting strategies were found in four CI participants, in that two participants relied more on loudness cues, and the other two participants relied more on pitch cues. The influence of loudness (or amplitude) contour on CI tone recognition has been demonstrated in several studies (Luo and Fu, 2004; Meng et al., 2016, 2018; Ping et al., 2017; Kim et al., 2021).

In this study, a much larger CI participant cohort was used to expand the findings, and more disyllables were carefully selected to generate an expanded speech corpus. The disyllable corpus includes five disyllabic words, which were decomposed and resynthesized into words whose primary (pitch) and secondary (loudness) cues varied independently. The syllables with flat tone were resynthesized to have either a high-flat, a rising, or a falling pitch contour. The pitch-manipulated monosyllables were then amplitude-modulated by three loudness gain functions, which are flat, rising, or falling. These resynthesized syllables formed a stimulus set of 270 disyllabic words (denoted by “Di”), each having a distinct meaning depending on the perceived tone. Thus, listeners who hear the primary pitch cue clearly will often hear a different meaning from listeners who are insensitive to the pitch cue and must rely on the secondary cue given by the loudness contour. The new stimulus sets thus make it possible to evaluate the contribution of pitch contour cues to lexical tone perception in CIs in isolation.

A tone recognition experiment was carried out with the new disyllabic set Di as well as with a set of natural monosyllabic recordings (“Mono”) (Wei et al., 2004) so that responses could be directly compared. The Mono stimuli consist of monosyllabic words with four tones which were recorded naturally from a female speaker. As noted before, natural Mandarin recordings contain pitch cues as well as co-varying secondary cues that can both help listeners identify lexical tones. In contrast, while Di includes only three tones (Tone 1, 2, and 4), its pitch contours and loudness contours were manipulated to vary independently, so that secondary loudness cues were no longer reliable, and pitch cue performance can therefore be assessed in isolation. In order to train the participants to use pitch contour as much as possible, participants were given trial-by-trial feedback of whether their answers were correct according to the pitch contour. Since pitch contour is the primary cue on which NH Mandarin speakers overwhelmingly rely for tone recognition, we scored a word as “correctly identified” when the listener reported the meaning of the word that corresponds to the lexical tone given by the pitch contour, irrespective of (secondary) loudness contour cues values.

## 2. Materials and methods

### 2.1. Participants

In total, seventeen CI recipients and eight NH listeners participated in this study. The CI recipients were recruited in Guangdong Province, and the NH listeners (age 18–32) were college students from two universities (South China University of Technology and Sun Yat-Sen University) in Guangdong Province. Further details about the CI recipients are shown in Table 1. The selection criteria for these CI participants were: (1) severe-to-profound sensorineural hearing loss in both ears, (2) more than 1-year CI use experience, (3) self-reported efficient speech communication ability without the use of visual cues, and (4) capable of cooperating to complete the experiment. Note that most of the participants were from Southern China, and some of them may use a Southern Chinese dialect to complete their day-to-day conversation with family members, such as Cantonese, so Mandarin may not have been their “mother tongue”. Participation was compensated and all participants gave informed consent in accordance with the Shenzhen University's ethical review board.

**Table 1:** Participant demographic and device information.

**Participant**	**Gender**	**Age range (yr)**	**CI experience (yr)**	**CI processor** **(R: Right; L: Left)**	**Etiology**
C21	F	31–35	7	R: Cochlear CP900	Drug-induced
C28	F	36–40	11	R: Cochlear N6	Ototoxicity
C30	F	21–25	1	R: Cochlear Freedom	Unknown
C34	M	11–15	13	R: Med-El OPUS2	Genetic
C36	M	16-20	L:4	L: Med-El OPUS2	Virus infection
			R:14	R: Med-El OPUS2	*Virus infection*
C37	M	11–15	10	L: Cochlear N6	Jaundice
C38	F	6–10	5	L: Med-El OPUS1	Pregnancy infection
C39	M	6–10	7	R: Cochlear N5	Unknown
C40	F	11–15	8	R: Cochlear Freedom	Unknown
C41	F	11–15	9	R: Cochlear CP900	Unknown
C42	M	21–25	18	R: Cochlear Freedom	Gentamicin allergy
C43	M	11–15	8	R: Med-El OPUS2	Ototoxicity
C44	F	31–35	8	R: Nurotron NSP560b	Progressive hearing loss
C45	M	11–15	10	R: Med-El OPUS2	Genetic
C46	F	21–25	1	L: Med-El OPUS2	Unknown
C47	F	16–20	10	L: Med-El OPUS2	Ototoxicity
C48	F	6–10	6	R: Med-El OPUS2	Ototoxicity

### 2.2. Stimuli

**Figure 1:**
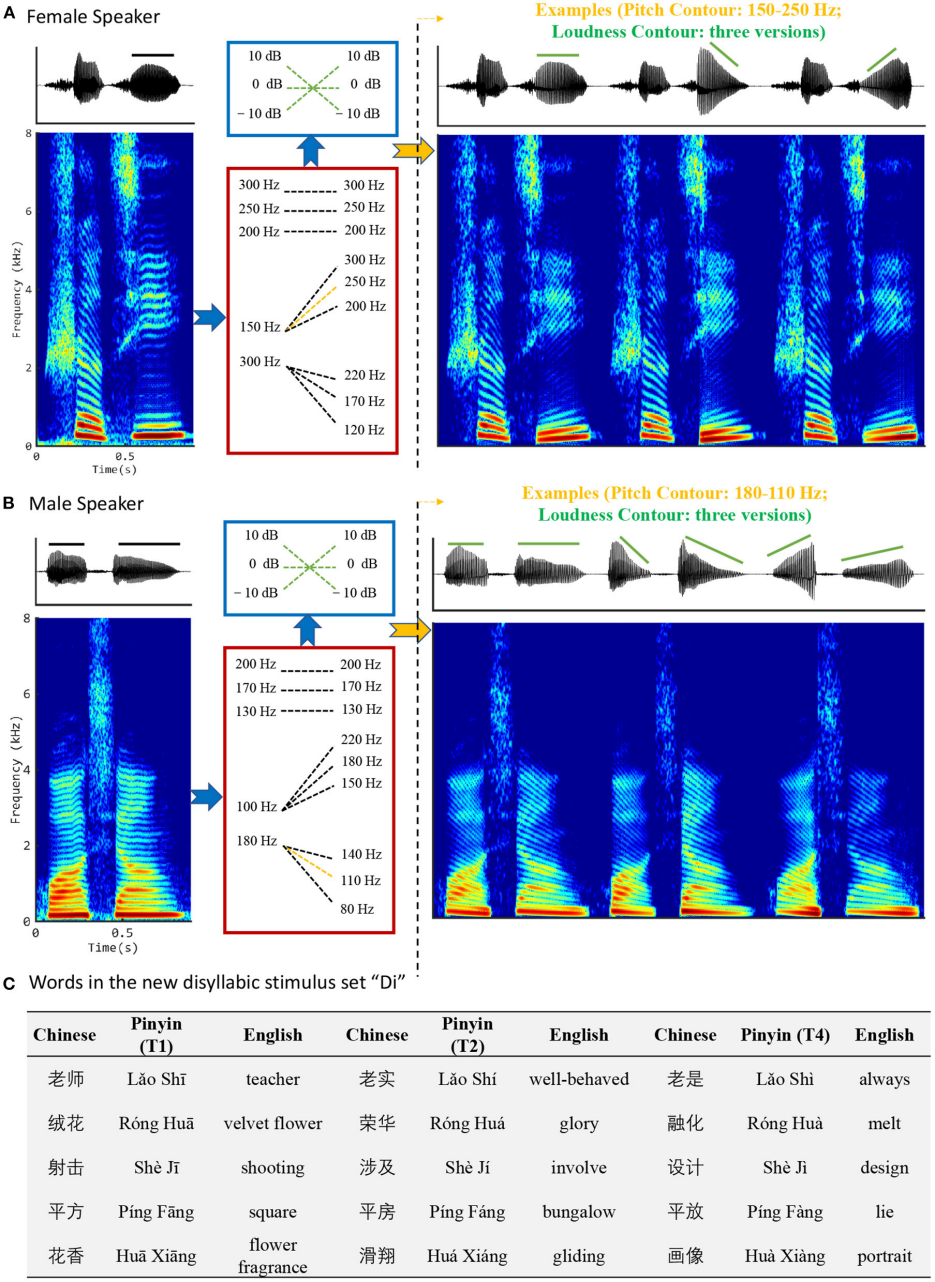
Illustrations of disyllabic stimulus generation. **(A)** The word /Shè Jī/ spoken by a female; **(B)** /Huā Xiāng/ spoken by a male. The left column shows the original waveforms and spectrograms. The middle column shows the strategies for manipulating the pitch and loudness contours. As shown, although the loudness-manipulation strategies are consistent for both male and female, the pitch-manipulation parameters are different between genders, reflecting the fact that females generally have higher pitched voices than males. The right column shows waveforms and spectrograms for /Shè Jī/ with a rising pitch contour (150–250 Hz) and three different loudness contours and /Huà Xiàng/ with falling pitch contours (180–110 Hz) and three different loudness contours. **(C)** Words in the new disyllabic stimulus set “Di”.

The new disyllables corpus consists of five main disyllabic words (i.e., /Lǎo Shī/, /Róng Huā/, /Shè Jī/, /Píng Fāng /, and /Huā Xiāng/), each recorded from 2 speakers (1 male and 1 female) in a studio at a sampling rate of 22,050 Hz and resampled using MATLAB resample.m to a sampling rate of 16,000 Hz. The STRAIGHT toolbox (17/09/2005) (Kawahara et al., 2004) was used to manipulate the pitch and loudness contours of the recorded signals. Firstly, the recorded words were decomposed according to a source-filter model to extract the excitation and spectral envelope related information. Then all the syllables with Tone 1 (i.e., the flat tone) were transformed to have 9 different F0 contours (changing linearly with time) including 3 flat contours, 3 rising contours, and 3 falling contours. Specific settings are shown in the Figure 1. For the female speaker, the 3 flat contours are 300, 250, and 200 Hz, respectively; the 3 rising contours are 150 to 300, 250, and 200 Hz, respectively; and the 3 falling contours are 300 to 220, 170, and 120 Hz, respectively. For the male speaker, the 3 flat contours are 200, 170, and 130 Hz, respectively; the 3 rising contours are 100 to 220, 180, and 150 Hz, respectively; and the 3 falling contours are 180 to 140, 110, and 80 Hz, respectively. These F0 values and frequency steps were selected with reference to the range of naturally recorded Chinese lexical tone frequency variations (Traunmuller and Eriksson, 1993; Moore and Jongman, 1997). The transformation was done by changing the F0 of the excitation signal accordingly and keeping the spectral envelope parts unchanged. This kept almost all information other than the pitch contour unchanged in the resynthesized signals. Finally, the amplitude of the voiced portion of each pitch-modified monosyllable was multiplied by three gain functions (i.e., 0 dB flat, −10 to +10 dB rising, and +10 to −10 dB falling) to generate different loudness contours. Figures 1A,B shows some examples of how the new disyllables were generated from the original recordings.

Permuting the 9 pitch contours with the 3 loudness contours, we generated 27 stimuli from each of the ten original disyllabic words (five for each gender), all having the same duration but differing independently in pitch and loudness contours. Thus, we obtained 270 stimuli (5 original disyllabic words × 2 speakers × 9 pitch contours × 3 loudness contours) in total. These 270 disyllabic stimuli formed our new Mandarin tone perception test stimulus set. Among the 270 disyllabic tokens, 90 tokens have the same pitch contours and loudness contours (both contours are high-flat, rising or falling, denoted by “Cov”), whereas the rest 180 have different pitch contour and loudness contours (denoted by “Conf”).

The synthesized syllables could be identified as one of the 15 disyllabic words shown in Figure 1C. It organizes them according to whether the second syllable has Tone 1, Tone 2, or Tone 4. Note that all the words created in this manner are common, easily understood, and easily distinguished words of Mandarin. Their English meanings are also shown in Figure 1C.

An existing stimulus set of naturally produced monosyllables (Wei et al., 2004) was used for comparison. It includes 100 tokens (25 monosyllabic words, each having four tone patterns) pronounced by a female speaker. For convenience, the disyllabic stimulus set generated in this study is noted as “Di” and the monosyllable set by Wei et al. (2004) is noted as “Mono”. Note that the Mono stimulus set consists entirely of unaltered recordings of naturally spoken Mandarin, and pitch and non-pitch cues to lexical tone will therefore naturally co-vary in the Mono stimulus set. In contrast, the Di stimuli are resynthesized so that pitch and loudness cues to lexical tone vary independently by design.

### 2.3. Procedure

**Figure 2:**
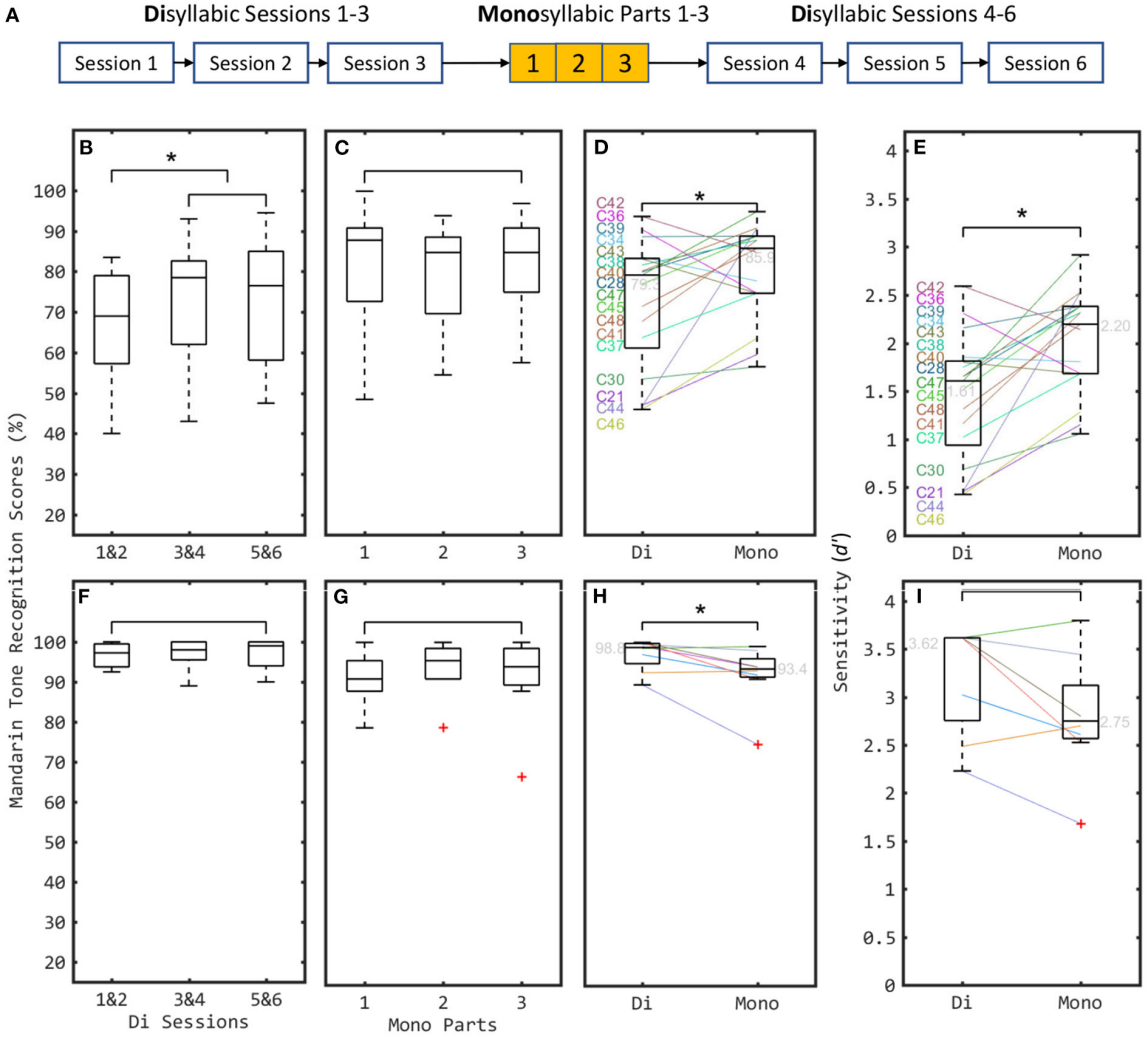
Mandarin tone recognition the new disyllabic stimulus set (denoted by “Di”) and the old monosyllabic set (denoted by “Mono”) for 17 CI **(B–E)** and 8 NH **(F–I)** participants. **(A)** Session procedure of experiment. Both Di sessions and Mono session were equally grouped or divided into three groups for analyzing the training effects of pitch-contour based correctness feedback. **(B,F)** Comparing results with three subgroups of Di. **(C,G)** Comparing results with three parts of Mono. **(D,H)** Individual results (shown by colored lines) and boxplots of the scores with Di and Mono stimulus sets, respectively. **(E,I)** Sensitivity index transferred from D&H. Significant differences between different conditions are illustrated by asterisks. Red plus signs indicate outliers, i.e., data exceeding 1.5 times the interquartile range. They are included in the formal analysis.

For each participant, the 270 Di stimuli were divided in a random order into 6 sessions, each with 45 stimuli. Between the third and fourth Di sessions, a test session with the 100 monosyllables from Mono was conducted, with all stimuli in a random order. The session order is shown in Figure 2A. The sound levels of all stimuli were normalized to have the same root-mean-square amplitude. Each stimulus was presented in one trial through an audio interface (Focusrite Scarlett 2i4) and a loudspeaker (Yamaha HS5I) at a sound pressure level of about 70 dBA in a sound-proof room. For the Di trials, a three-alternative-forced-choice (3AFC; T1, 2, or 4) task was used; for the Mono trials, a 4AFC was used (T1, 2, 3, or 4). In each trial, three or four buttons including the Chinese characters and Mandarin Pinyin were shown in a graphic user interface for the subjects to select using a mouse, and the correctness of the subject's choice (according to the pitch-tone) was shown as green (correct) and red (incorrect) colors in another user interface element.

### 2.4. Statistical analysis methods

A Wilcoxon signed rank test was used to compare within-subject conditions; a Wilcoxon rank sum test was used to compare between-subject conditions; a Holm-Bonferroni correction was used for multi-pair comparison; and a Spearman's rank correlation analysis was used to quantify correlations between performance and CI hearing experience. In the result figures, the raw percentage correct scores are shown for simplicity, but to make the results from Di 3AFC and those from Mono 4AFC tests quantitatively comparable, irrespective of their differing chance % correct levels, sensitivity index (d-prime, *d*′) values were computed and statistical tests were carried out on the *d*′ values. The dprime.mAFC function from the psyphy library of the R programming language was used for this conversion. The mapping between percentage scores and *d*′ can also be found in Hacker and Ratcliff (1979).

## 3. Results

### 3.1. Training effects

Feedback was given in each trial based on whether the response was correct according to the pitch cue of the stimulus. This encouraged the subjects to use pitch-contour information to do the task. For the CI subjects, the median scores pooled over Di Sessions 1 & 2 were significantly lower than those for Sessions 3 & 4 (*Z* = −2.771, *p* < 0.01, *n* = 17, Wilcoxon signed rank test) and 5 & 6 (*Z* = −2.699, *p* < 0.01, *n* = 17). No significant difference was found when comparing the pooled median scores from Di Sessions 3 & 4 against 5 & 6 (*Z* = −0.466, *p* = 0.641). Also, no significant difference was found between the median scores obtained with three parts of Mono (*Z* = −1.434, −0.035 and −1.846, respectively, *p* > 0.05 for all comparisons, *n* = 17) (see Figures 2B,C). For the NH subjects, the median scores between three subgroups of Di and between three parts of Mono showed no significant difference (*Z* = −2.521, −0.542, 0.000, −1.511, 0.000, and −1.121, respectively, *p* > 0.05 for all comparisons, *n* = 8) (see Figures 2F,G). Therefore, a significant training effect was found over the first two sessions of Di with CIs. The performance reaches a ceiling from session 3 onwards. Consequently, the results from Di Sessions 3, 4, 5, and 6 were pooled to compute the performance scores for both CI and NH cohorts in the Di task.

### 3.2. CI vs. NH

**Figure 3:**
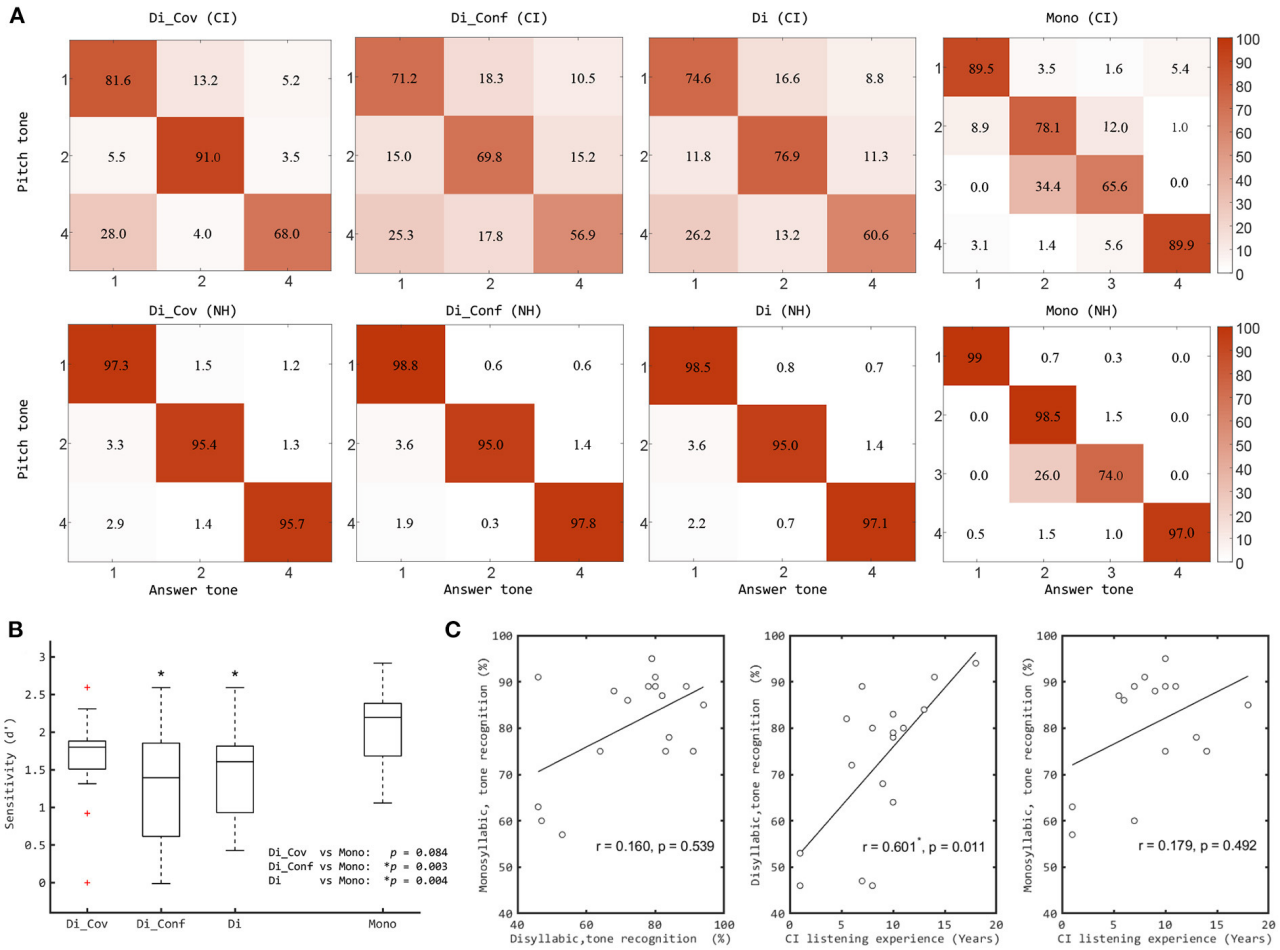
**(A)** Confusion matrices for pitch tone in CI and NH listeners using different corpus (the number represents the tone recognition percentage scores). **(B)** Boxplots of Mandarin tone recognition percentage scores of CI listeners based sensitivity index with sub-conditions (Cov and Conf) of “Di” conditions, compared with “Mono” conditions. The significant differences between Di conditions and Mono are illustrated by the asterisks. **(C)** Correlations between Mandarin tone recognition scores with “Mono” and “Di” and the CI participants' listening experience. The significant correlation is marked by an asterisk. Di, the new disyllabic stimulus set; Mono, the old monosyllabic stimulus set; Cov, covarying pitch and loudness contours; Conf, conflicting pitch and loudness contours. Red plus signs indicate outliers (like that in Figure 2).

The Mandarin tone recognition scores for both Di and Mono stimulus sets are summarized in Figures 2D,H. The median scores of the CI participants (79.3% for Di and 85.9% for Mono) were significantly lower than those of the NH participants (98.8% for Di and 93.4% for Mono) [*Z* = −2.521 (Di) and −2.240 (Mono), *p* <0.05 for two comparisons, *n* = 25, Wilcoxon rank sum test, Holm-Bonferroni corrected]. NH listeners recognized the words from both stimulus sets with general good scores (see Figures 2H, 3A). The only difficulty for NH with Mono is they sometimes (26.0%) identified the Tone 3 as Tone 2. For Di, Tone 3 was not included, so this confusion was not examined. What's more, in the Di stimulus set, where pitch and loudness cues often diverged, the primary cue (pitch) clearly dominated for NH listeners, as NH listeners were hardly ever misled by conflicting loudness cues. In contrast, CI users scored more poorly, particularly in the tests involving the Di speech material, where accurate pitch coding is particularly important.

### 3.3. Di vs. Mono

Indeed, for the CI cohort, the median performance with Di (79.3%, *d*′ = 1.61) was significantly lower than with Mono (85.9 %, *d*′ = 2.02) (*Z* = −2.911, *p* = 0.004, *n* = 17, Wilcoxon signed rank test on *d*′ values, see Figure 2E). In contrast, for the NH cohort, the median score with Di (98.3%) and the scores of most (6/8) participants was higher than those with Mono (see Figure 2H), even though this median *d*′ difference was not statistically significant (3.62 with Di, and 2.75 with Mono) (*Z* = −1.823, *p* > 0.05, *n* = 8, see Figure 2I. Thus, Di was more difficult than Mono for CI users, as expected given the at times conflicting secondary cues.

### 3.4. Dominant cues for CIs

In Figures 3A,B, we also show the results of CI listeners using the disyllabic stimuli subdivided according to whether the pitch and loudness cues were “Covarying” or “Conflicting”. CI users performed significantly better in covarying conditions than in conflicting conditions (see Figure 3A). The median score in the covarying condition was significantly higher than that in the conflicting condition (*Z* = −2.215, *p* < 0.05, *n* = 17, Wilcoxon signed rank test) (Figure 3B). When comparing Mono with the subgroups of Di, the median score with Mono was significantly higher than the score for the Conflicting (*Z* = −3.006, *p* < 0.05, *n* = 17, Wilcoxon signed rank test, Holm-Bonferroni corrected) but did not differ significantly from the Covarying stimulus trial results (*Z* = −1.728, *p* > 0.05). These results indicate that secondary cues were used by most CI users for tone recognition.

### 3.5. Correlations with CI listening experience

Figure 3C shows the correlations between the tone recognition scores with two stimulus sets and the CI subjects' listening experience. No significant correlation was found between the tone recognition scores obtained with the two stimulus sets (*r* = 0.160, *p* = 0.539, Spearman's rank correlation analysis). With the Mono stimulus set, no significant correlation was found between tone recognition results and CI listening experience (*r* = 0.179, *p* = 0.492). With Di stimulus set, however, a highly significant correlation was found between tone recognition results and the amount of CI listening experience (*r* = 0.601, *p* = 0.011). In the CI cohort, subjects with longer experience generally also have an earlier implantation age. A significant (albeit somewhat weaker) correlation was also found between tone recognition results with Di and their implantation ages (*r* = −0.537, *p* = 0.026).

## 4. Discussion

Many studies have shown that Chinese CI users have reasonably good Mandarin tone recognition in quiet environments, usually higher than 60% on average, and higher than 90% for some star participants (Wang et al., 2011, 2012; Tao et al., 2015; Gu et al., 2017; Mao and Xu, 2017; Vandali et al., 2017; Li et al., 2018). However, all these experiments used stimulus sets of naturally produced sound recordings, in which secondary cues, such as loudness contour and syllable duration, can also contribute to a CI user's tone recognition, and pitch contours are not the only cues. Therefore, it is hard to attribute a CI participant's performance in Mandarin tone recognition specifically to the strengths or weaknesses of their pitch encoding, even if pitch cues are generally acknowledged to dominate tone perception in NH listeners (a fact also confirmed in this study). Furthermore, multiple cues may lead to ceiling effects in performance, which make it difficult to evaluate the effectiveness of pitch-based tone enhancement strategies (Vandali et al., 2017).

Our new disyllabic stimulus set isolates pitch cues from secondary cues by eliminating duration cues and varying amplitude contour cues orthogonally to pitch cues. Results from CI users revealed a substantial drop in median tone recognition scores when they were tested with our new stimuli in comparison to the existing monosyllabic stimulus set in which multiple cues covaried (see Figure 2D). The tone recognition scores with both Di and Mono were much higher for NH listeners than for CI users. This indicates that considerable shortcomings remain in the encoding of pitch cues for tone recognition through CIs. Furthermore, the tone recognition performance of CI users was better when secondary cues co-varied with the pitch cues compared to when these varied independently. This discrepancy was not found in NH listeners (see Figure 3A). These observations can be explained if we assume that the pitch and amplitude cues to Mandarin tone are weighted differently in NH and CI listeners. While NH listeners appear to rely on pitch cues almost exclusively, some CI users who have difficulty using pitch cues (i.e., poor tone recognition in Conf conditions) may learn to rely more on secondary cues instead. The fact that in the DI corpus pitch and secondary cues vary independently makes it possible to determine the extent to which individual CI users are able to rely on primary pitch or secondary loudness contour cues respectively when attempting to identify lexical tones.

Furthermore, the scores with the new stimulus set correlated strongly and significantly with the CI participants' implantation ages and listening experience, in contrast with the scores obtained with the older stimulus set which conflates multiple cues, and which therefore cannot accurately assess the users' ability to discriminate pitch cues for tone recognition. Thus, the ability to utilize pitch cues for tone recognition tasks improves both with earlier implantation and longer hearing experience with CIs (see Figure 3C). However, NH listeners recognized the new disyllabic tones more accurately than the monosyllabic tones, which might benefit from the context of pitch between the two syllables, and the removal of the Tone 3 (falling-then-rising), which is easily confused with Tone 2 (rising) (Figure 3A). In addition, the naturalness of the stimuli could perhaps be somewhat compromised by the fact that the tones of the disyllabic words are synthetic. However, the STRAIGHT method used is generally capable of synthesizing quite naturally sounding speech samples. Interested readers familiar with the sound of Mandarin can of course download the Di speech samples from the github repository and judge for themselves how natural they sound. In any event, the fact that NH cohorts were able to score very highly with the Di corpus, and no worse than with the Mono corpus which consisted of natural recordings (Figures 2F,G), suggests that the naturalness of the Di stimuli is at least adequate to facilitate highly accurate word recognition among native Mandarin speakers, giving confidence that the stimuli are adequate for the intended purpose in audiological assessment.

An important application of the new Di stimulus sets is to reduce the confounds and ceiling effects that can be caused by the secondary cues, and which can plague the evaluation of some tone enhancement strategies (Vandali et al., 2017). In the light of our findings, it seems likely that CI users with poorer pitch coding may compensate by weighting loudness and duration cues more heavily, which would mask the true extent of their pitch coding deficits. Some authors have sought to reduce the ceiling effects by adding noise (Wei et al., 2004; Gu et al., 2017; Mao and Xu, 2017; Vandali et al., 2017). Understanding speech in noise is a challenge that both NH and CI listeners often have to contend with. However, the addition of noise may mask both pitch and loudness contour cues in complex ways that will depend on the type of background noise and may be hard to predict. It would therefore be very useful to conduct speech-in-noise recognition experiments with stimulus sets like the one developed here, which make it possible to study the relative effects of noise on pitch and loudness cue processing for lexical tone independently.

## 5. Conclusion

A new Mandarin tone corpus consisting of five main disyllabic words from two speakers was developed in this study. In this corpus, there is no reliable secondary cue that could be used by listeners to facilitate the pitch-contour based tone recognition (i.e., loudness contours change independently of pitch contours). When compared to NH listeners, CI users had poorer pitch cue encoding, and their tone recognition performance was significantly influenced by the “missing” or “confusing” secondary cues with this corpus. The corpus could be used to examine the performance of pitch recognition of CI users and the effectiveness of pitch cue enhancement based Mandarin tone enhancement strategies. Listeners with longer CI listening experiences tend to get higher scores of tone recognition with this corpus.

## Data availability statement

The raw data supporting the conclusions of this article will be made available by the authors, without undue reservation.

## Ethics statement

The studies involving human participants were reviewed and approved by Shenzhen University's Ethical Review Board. Written informed consent to participate in this study was provided by the participants' legal guardian/next of kin.

## Author contributions

QM and YZ contributed to conception and design of the study. XW, FK, and WG carried out the experiment and organized the database. YM and HZ performed the statistical analysis. XW and YM wrote the first draft of the manuscript. NZ and JS wrote sections of the manuscript. All authors contributed to manuscript revision, read, and approved the submitted version.

## Funding

This work was supported by the Guangdong Basic and Applied Basic Research Foundation Grant (2020A1515010386 and 2022A1515011361) and Science and Technology Program of Guangzhou (202102020944).

## Conflict of interest

The authors declare that the research was conducted in the absence of any commercial or financial relationships that could be construed as a potential conflict of interest.

## Publisher's note

All claims expressed in this article are solely those of the authors and do not necessarily represent those of their affiliated organizations, or those of the publisher, the editors and the reviewers. Any product that may be evaluated in this article, or claim that may be made by its manufacturer, is not guaranteed or endorsed by the publisher.
